# Care quality and satisfaction at the cancer hospital – a questionnaire study of older patients with cancer and their family members

**DOI:** 10.1186/s12913-024-10646-4

**Published:** 2024-02-12

**Authors:** Minna Launonen, Katri Vehviläinen-Julkunen, Santtu Mikkonen, Tarja Kvist

**Affiliations:** 1 Department of Nursing Science, University of Eastern Finland, Kuopio, Finland; 2 Kuopio University Hospital, Kuopio, Finland; 3 Faculty of Science, Forestry and Technology, Department of Technical Physics, University of Eastern Finland, Kuopio, Finland

**Keywords:** Older patients with cancer, Family member, Quality care, Satisfaction, Acute care

## Abstract

**Background:**

The unique life situations of older patients with cancer and their family members requires that health care professionals take a holistic approach to achieve quality care. The aim of this study was to assess the perceptions of older patients with cancer and family members about the quality of care received and evaluate differences between their perceptions. A further aim was to examine which factors explain patients’ and family members’ levels of satisfaction with the care received.

**Methods:**

The study was descriptive and cross-sectional in design. Data were collected from patients (*n* = 81) and their family members (*n* = 65) on four wards in a cancer hospital, using the Revised Humane Caring Scale (RHCS). Data were analysed using descriptive statistics, crosstabulation, Wilcoxon signed rank test, and multivariable Analysis of Covariance (ANCOVA).

**Results:**

Family members had more negative perceptions of the quality of care than patients did. Dissatisfaction was related to professional practice (*p* < 0.001), interaction between patient and health care professionals (*p* < 0.001), cognition of physical needs (*p* = 0.024), and human resources (*p* < 0.001). Satisfaction with overall care was significantly lower among those patients and family members who perceived that they had not been involved in setting clear goals for the patient's care with staff (*p* = 0.002).

**Conclusions:**

It is important that older patients with cancer and family members receive friendly, respectful, individual care based on their needs and hopes, and that they can rely on professionals. Health care professionals need more resources and education about caring for older cancer patients to provide quality care.

## Background

It is estimated that a quarter of the European population will be over 65 years of age by 2050 [1]. Globally, the incidence of new cancer cases is rapidly growing, at 18.1 million in 2020 and expected to reach 28–30 million by 2040 [2, 3]. On average, half of these will be diagnosed in people over 65 years of age [3]. This age group is very heterogeneous in terms of morbidity and cognitive disorders. Some older patients with cancer have complex needs, while others stay in good health [4, 5]. Addressing their various unique needs is costly, especially as resources are often inadequate in cancer care [5–7].

The main need of older patients with cancer is the maintenance of their independence and freedom. Physical mobility is important for performing the activities that give them joy and satisfaction in life [8]. In addition, needs based on the individual’s personal circumstances, preconceptions, and knowledge about cancer affect their experience regarding the quality of care [6]. Furthermore, satisfaction is an important indicator of care quality, and measuring it provides insights into how well patients’ care needs have been fulfilled [7].

Quality of care is a human right regardless of age. Various countries in Europe have charters, specific laws, or administrative regulations about patients’ rights, most of which mention quality of care [9]. The World Health Organization (WHO) and the Institute of Medicine (IOM) define quality of care in terms of efficiency, safety, patient-centredness, timeliness, equity, integration, and efficiency [7, 10]. In recent years, this definition has expanded to focus more on patient perspectives, psychological aspects, and care planning, as well as on meeting the needs of patients and their families [11], since older patients with cancer and their family members together face the challenges of different stages of cancer treatment, including other challenges related to heath and everyday life [6]. Nevertheless, while family members of older patients have expressed their will to be involved in care during the patient’s hospital stay, they have faced several challenges [12]. It has previously been reported that older patients with cancer have fewer unmet needs than younger patients [13]; however, they may have difficulties expressing their needs to health care professionals [14], or they may hesitate to ask for help when needed [6].

While there is some literature exploring the experiences of older patients and their families in acute care [13], there is little research that focuses specifically on older patients with cancer, and this research has been limited to the contexts of ambulatory [15] and palliative care [16]. Providing quality care is a challenge, and it is important to understand the perceptions that older patients with cancer and their families have of both the quality of care and their satisfaction with it [5, 17]. Therefore, the purpose of this study is to focus on patients’ and family members’ individual perspectives on the process of care provision, as this has been found to be a better measure of care quality and satisfaction than the outcomes of care [7].

## Methods

### Aim

This study aims to assess how older cancer patients and their family members perceive the quality of the care they are receiving and to evaluate the differences between their perceptions. A further aim is to examine which factors explain patients’ and family members’ levels of satisfaction with the care received. It thus addresses the following research questions:

1. How do older cancer patients and their family members perceive the quality of care?
2. Are there differences between the perceptions of older cancer patients and those of their family members with regard to the quality of care?
3. Which factors explain patients’ and family members’ satisfaction with the care received?

### Design, setting, and sample

This study is quantitative, descriptive, and cross-sectional in design, and the participants are part of a larger study. Data were collected from a 78-bed cancer hospital providing acute care to older patients with cancer. The inclusion criteria were as follows: patients were aged 65 or more, had cancer, and were fluent in Finnish. The inclusion criteria for family member were that they were participating in the patient’s care, including at home, and were fluent in Finnish. Age 65 was selected as the threshold age for patients in order to align with most wealthy countries’ definitions of ‘old’ [18].

The minimum sample size to detect the expected effect size of 0.3 would be 111. We did not achieve this, but the response rates (40.5% for patients, 32.5% for family members) were deemed to be acceptable. We delivered 200 questionnaires to patients and 200 questionnaires to family members to the wards, and 81 patients and 65 family members completed the questionnaires.

### Data collection

Data collection was carried out between October 2016 and May 2018 using convenience sampling. Recruitment was organized by the researcher, nurses, and two contact persons in the cancer hospital. Information about the study protocol was presented to the nurses during meetings in October 2016 and January 2018. Written information about the study protocol was also provided, including the researcher’s contact information for any enquiries.

Paper questionnaires were handed to patients on the ward. They could either complete the questionnaire on the ward or at home after discharge, returning it to the researcher using the pre-paid envelope provided. The patients were also given a questionnaire to pass on to a family member. Written information about the study was provided alongside the questionnaire, and written informed consent was sought from both patients and family members.

### Instruments

The Revised Humane Caring Scale (RHCS) was used to measure quality of care and satisfaction. This includes 42 items, organized under six headings, which are measured using a 5-point Likert scale from 1 (full disagreement) to 5 (full agreement). The headings consist of professional practice (17 items), information and participation in own care (11 items), cognition of physical needs (4 items), human resources (3 items), pain and apprehension management (4 items), and interdisciplinary collaboration (3 items). The instrument also includes two outcome variables: *‘A clear goal for care is set by me, family members and staff, together’* and *‘I am satisfied with my care’* for patients and *‘A clear goal for care is set by the patient, me as a family member, and staff, together*’ and *‘I am satisfied with my family member’s care’* for family members [19]. The RHCS has been used since the 1990s in various nursing contexts. Cronbach’s alpha values were between 0.640 and 0.937, and they have previously been reported as between 0.775 and 0.970 [20, 21].

### Data analysis

Descriptive statistics (frequencies and percentages) were used to describe older cancer patients and their family members, while means and standard deviations (SD) were used to represent continuous variables (Table 1). In the analysis, the respondents’ ages were organized into categories, and the domains of the RHCS and the outcome variables were transformed from 5-point Likert scale responses into dichotomous responses (disagree = fully disagree, disagree, and cannot say/unsure vs. agree = fully agree and agree). Cross-tabulation and a chi-square test were used to detect differences between older patients with cancer and family members regarding the quality of care (Table 2).

**Table 1:** Descriptive characteristics of older patients with cancer (*n* = 81) and family members (*n* = 65) (n, %)

	Patients n (%)	Missing n (%)	Family members n (%)	Missing n (%)
**Gender**				
Female	43 (53.1)		46 (70.8)	0 (0.0)
Male	38 (46.9)	0 (0.0)	19 (29.2)	
**Age**				
≤ 70	40 (49.4)		44 (67.7)	1 (1.5)
> 70	38 (46.9)	3 (3.7)	20 (30.8)	
	(Mean ± SD: 72 ± 5.42)		(Mean ± SD 63 ± 12.98)	
**Health status**				
Good	23 (28.4)		45 (70.0)	1 (1.5)
Not good	56 (69.1)	2 (2.5)	19 (29.2)	
**Cancer**				
Gastrointestinal	8 (9.9)			
Urological	10 (12.3)	3 (3.7)		
Gynecological and breast cancer	28 (34.6)			
Hematological cancer	24 (29.6)			
Other^a^	8 (9.9)			
**Professional education**				
Academic degree	37 (45.7)	2 (2.5)	26 (40.0)	1 (1.5)
Vocational education	24 (29.6)		30 (46.2)	
No professional education	18 (22.2)		8 (12.3)	

Data are expressed as means ± SD for continuous variables and as number and percentage for different categorical variables

*SD* Standard Deviation, *n* Number

^a^pancreatic and peritoneal cancer, sarcoma

**Table 2:** Comparison between older patients with cancer (*n* = 81) and family members’ (*n* = 65) perceptions of care quality

RHCS: variable^a^	Older patient with cancer(*n* = 81)		Family members (*n* = 65)		*p*-value
	Disagree %	Agree %	Disagree %	Agree %	
I/the patient was accepted	2.5	97.5	14.3	85,7	0.011
My/the patient’s concerns are listened to when I/she/he have worries	3.7	96.3	28.6	71.4	< 0.001*
The right level of interest is shown for me/the patient	2.5	97.5	22.2	77,8	< 0.001*
My/the patient’s assessment of how I/she/he feel is relied upon	7.4	92.6	28.6	71.4	0.001
I/the patient can discuss issues with nurses in confidence if necessary	6.2	93.8	27.0	73.0	< 0.001*
I/the patient can speak with the staff in private	22.2	77.8	39.7	60.3	0.019
I am/the patient is welcomed onto the ward	2.5	97.5	14.3	85.7	0.011
I/the patient feel safe on the ward	1.2	98.8	14.3	85.7	0.005*
I/the patient can ask questions concerning my/her/his care	3.7	96.3	17,5	82.5	0.009
I am/the patient is given clear information about instructions and restrictions related to treatments	3.7	96.3	19.0	81.0	0.005
I/the patient receive enough information about my/her/his follow-up treatment	13.6	86.4	30.2	69.8	0.022
I am/the patient is addressed in clear and intelligible language	1.2	98.8	14.3	85.7	0.005*
My/the patient’s state of health is inquired sufficiently	1.2	98.8	28.6	71.4	< 0.001*
I receive/the patient receives help when I/she/he need it	2.5	97.5	12.7	87.3	0.021
I am/the patient is treated with respect	1.2	98.8	11.1	88.9	0.021
I am/the patient is treated in a friendly manner	2.5	97.5	11.1	88.9	0.042
My/the patient’s fears are alleviated	35.0	65.0	61.9	38.1	0.002
The staff have enough time for me/the patient family member	3.7	96.3	30.2	69.8	< 0.001*
There are enough members of staff on the ward	8.6	91.4	33.3	66.7	< 0.001*
The atmosphere on the ward is unhurried	8.6	91.4	42.9	57.1	< 0.001*
The on the ward is positive	3.7	96.3	42.9	57.1	< 0.001*
The nursing staff act professionally	1.2	98.8	19.0	81.0	< 0.001*

Disagree = fully disagree + disagree + cannot say/unsure vs. agree = fully agree + agree

^a^Answers depending on who is completing the questionnaire: I/my (patient), the patient (family member)

^*^Represent significant associations determined by Chi-square test, *p*-values < 0.05 are considered statistically significant (Fishers exact test)

The normal distribution of the subscales of the RHCS was tested using the Kolmogorov–Smirnov test. Because this test showed that the subscales were not normally distributed, the non-parametric Wilcoxon signed-rank test was used to detect differences between older patients with cancer and family member pairs regarding the quality of care (Table 3).

**Table 3:** Differences in perceptions about the quality of care between 56 patient-family member pairs (*n* = 112)

Subscale	Cancer patient	Family member	*P* value*
	Mean ± (SD)	Mean ± (SD)	
Professional practice	4.80 ± (0.257)	4.39 ± (0.570)	< 0.001*
Information and participation in own care	4.48 ± (0.435)	4.07 ± (0.661)	< 0.001*
Cognition of physical needs	4.33 ± (0.820)	4.08 ± (0.748)	0.024*
Pain and apprehension management	3,85 ± (0.679)	3.69 ± (0.661)	0.067
Human resources	4.58 ± (0.700)	3.87 ± (0.872)	< 0.001*
Interdisciplinary collaboration	4.58 ± (0.639)	4.37 ± (0.709)	0.057
Total mean score	4.44 ± (0.36)	4.07 ± (0.55)	< 0.001*

Data are expressed as means ± SD for continuous variables

*SD* Standard Deviation

^*^Wilcoxon signed rank test, *p*-values < 0.05 are considered statistically significant

ANCOVA was used to examine the differences in the mean values for satisfaction with care, adjusting for the effect of other variables. To do this, the data for older patients with cancer and family members were combined into one dataset. Satisfaction with care was set as the dependent variable, the RHCS subscales were applied as covariates, and background characteristics and the outcome variable *‘A clear care goal is set together’* were applied as independent variables. From the data, seven models of the satisfaction with care of older patients with cancer and family members were constructed (Table 4). For the analysis of covariance, the normal distribution of the residuals was checked by visual inspection of the histograms. For all of these analyses, the level of significance was set at 0.05 [22], and the data were analysed using SPSS® version 27.00 for Windows [23].

**Table 4:** The relationship between cancer patients and family members’ characteristics, RHCS and satisfaction with care (*n* = 133)

Dependent variable: Satisfaction with care														
	Covariates													
Independent variables*	Total mean score of RHCS		Professional practice		Information and participation in care		Pain and apprehension management		Cognition of physical needs		Human resources		Interdisciplinary collaboration	
	B	p**	B	P**	B	p**	B	p**	B	p**	B	p**	B	p**
Patient (ref. family)	0.398	0.001	0.278	0.020	0.435	< 0.001	0.491	< 0.001	0.486	< 0.001	0.392	0.002	0.464	< 0.001
Disagreement about the clear care goal (ref. agreement)	-.924	0.002	-0.846	< 0.001	-0.952	< 0.001	-0.983	< 0.001	-0.989	< 0.001	-0.977	< 0.001	-0.930	< 0.001

*Abbreviations*: *RHCS* Revised Humane Caring Scale, B Unstandardized coefficient

^*^Significant results in ANCOVA, *p*-values < 0.05 are considered statistically significant and presented

^**^Adjusted for age, gender, health status, education, perceptions about ‘*A clear goal for the care is set together’,* and group (patient or family member)

## Results

The age of 81 older patients with cancer Mean ± SD was 72 ± 5.42, and the age of 65 family members Mean ± SD was 63 ± 12.9. More than half of the patients (53%) and two thirds of the family members (71%) were female. A majority of the older patients with cancer (75%) and family members (86%) had some professional education. Perceived health was reported as good by 70% of family members but only 28% of patients (Table 1).

### Perceptions of care quality of older patients with cancer and family members

The perceptions of older patients with cancer and family members about the quality of care were good: the mean value of their responses to the subscales of the RHCS varied between 2.97 and 4.95 (range: 1 = lowest, 5 = highest). Patients attached most value to their treatment being carried out with respect (4.95) and friendliness (4.95) and to receiving help when needed (4.91). However, patients were less satisfied with their participation in care planning (3.75) and receiving pain relief through non-pharmacological methods (2.92). Family members perceived that their loved one’s pain was taken seriously (4.71), they were treated with friendliness (4.62), and their care was safe (4.60). They were dissatisfied with the use of non-pharmacological pain relief (2.97). They also felt that patients’ fears were not alleviated (3.54) and that they, as family members, did not have enough opportunities to take part in care planning (3.49).

The results revealed some differences between the assessments of older patients with cancer and of family members. For over half of the RHCS items, patients and family members gave statistically significantly different responses about the domains of care quality. In all of these instances, patients assessed their care more positively than family members did. The statistically significant results are presented in Table 2.

### Differences in perceptions about the quality of care between patient and family member pairs

In total, 56 older patients with cancer and family member pairs (*n* = 112) were investigated. Statistically significant differences between paired patients and family members were observed for all six subscales and the total mean score of the RHCS. In order to form a pair, the patient had to have a person classified as a family member to whom they could give the questionnaire. The results show that family members’ assessments of the quality of care were lower (4.07) than that of patients (4.44) (Table 3).

### Factors explaining satisfaction with care

We examined the statistical difference between the outcome variable *‘Satisfaction with care’* and the RHCS, when controlling for age, gender, health status, education, the outcome variable *‘A clear care goal is set together’*, and group (patient or family member). After controlling for the total mean score of the RHCS, perception about the variable *‘A clear care goal is set together’* (F (2,127) = 13.608, *p* < 0.001, η^2^ = 0.176) and participant group (F (1,127) = 10.189, *p* = 0.002, η^2^ = 0.074) had a relationship with satisfaction with care. Those who disagreed with having a clear goal of care perceived scores nine points lower than those who agreed (B = -0.924), and patients perceived scores three points higher than family members (B = 0.398). Exploring the subscales separately revealed similar results. Both independent variables together accounted for approximately 35% of the variance in satisfaction with care (R^2^ = 0.357) (Table 4.)

## Discussion

This was a cross-sectional descriptive study of older cancer patients who have rarely been studied in the context of acute care in a cancer hospital. The study also provided insight into their family members’ experiences during the challenging time that patients spent in acute care.

### Patients’ perceptions of the quality of care

We found that older cancer patients described some difficulties with their participation in care planning. One explanation for this may be that older cancer patients have cognitive impairments [1] that health care professionals may be unaware of, leading to problems and misunderstandings [12]. Second, nurses have described that elderly patients do not tell them what they want [6, 14], also causing misunderstandings. Nevertheless, earlier studies have shown that it is important that patients participate in care planning and receive adequate explanations of their treatment goals and the future [5, 11].

In this study, patients perceived that the care they received was friendly and respectful. Kind and friendly behaviour by nursing professionals towards patients has been shown to improve the experience of care quality among older cancer patients in hospital settings [24], and patients have described that respectful attitudes have a significant influence on their experience of care [25]. Respectful care has also been previously associated with patients’ needs [26]. Our results showed that patients described their needs for help being fulfilled during their hospital stay, and previous research supports this result by showing that older people have fewer unmet needs [14]. It must be remembered that, as older patients may have diseases that limit their comprehension and affect their daily lives, getting help when needed is especially essential to their perception of whether or not care is satisfactory [27]. Sensitivity in meeting and interpreting the needs of older patients with cancer is essential [12]. However, it has previously been shown that nurses may find it difficult to manage older patients’ basic needs due to a lack of staff and time [14, 28], meaning that adverse events occur, and care quality is undermined [29].

### Family members’ perceptions of the quality of care

Family members expressed that the patient’s pain was taken seriously. This suggests that patients in this study received sufficient pain medication. However, nurses should nonetheless remain alert, as it has been shown that older patients may hide their pain or report it less than younger patients do [30]. This may be because they feel that revealing pain shows weakness, or they might be afraid of painkillers causing addiction [31].

While family members were satisfied that patients were taken seriously when they were in pain, they also said that non-pharmacological pain management methods were deficient. It is possible that some such methods – for instance, kinesiotherapy – may not be visible to family members because they are limited to visiting the hospital during restricted visiting hours [12].

In this study, family members valued the friendly way in which patients were treated. Family members appeared to draw comfort from this, particularly in acute situations where they were in daily contact with patients who were experiencing distress [30]. Family members also felt that patient care on the ward was safe. Previous studies have shown that patients feel safe when nurses visit them very frequently, even if they are busy, but less so if the nurses do not listen but just carry out their tasks and then leave [12].

However, family members were concerned that patients’ fears were not alleviated. There is a chance that this actually reflects poor communication between patients and their family members [32] – not telling relatives what they need [14]. It is also likely that family members themselves have their own fears about the patient’s illness and deterioration [33] and were reporting these fears rather than those of the patient [34].

Family members also reported not having enough opportunities to participate in care planning, aligning with a similar result that had been discovered earlier [14]. This may be due to limited contact with nurses, which makes it difficult to participate in care planning [35]. Perceptions of care quality have previously been shown to depend strongly on having clear information about the next step in care and follow-up [36].

### Differences between patients’ and family members’ perceptions of the quality of care

We found significant differences between patients’ and family members’ perceptions of the quality of care, with family members giving a more negative assessment than patients. Family members were primarily concerned about a lack of resources for patient care, as well as the negative, rushed atmosphere on the ward. The number of nurses and the working environment have a direct impact on the satisfaction and quality of care [20, 37].

It has previously been shown that nurses prioritize their activities during staff shortages to guarantee patient safety. They prioritize their time to perform vital symptom assessments or administer medication at the expense of bathing patients, carrying out skin care, or ambulation [28]. Moreover, the basic physical needs of the patients are easier to fulfil than their psychological needs [14]. However, sometimes even essential nursing tasks are found to be left undone due to lack of staff [29].

Family members described that there is a lack of communication between patients and staff. They felt that the staff did not enquire enough into the patients’ state of health – the right level of interest was not shown, their worries were not listened to, and it was not possible to arrange discussions in confidence. Caring for older patients is challenging, causing emotional stress to staff, and sometimes keeping a certain distance from patients can relieve this stress. In general, nurses have positive attitudes towards older patients and do not deliberately compromise their professional or ethical principles when dealing with them [38]. However, there is evidence to suggest that such compromises and negative attitudes sometimes do arise [14, 39].

Finally, our results show a slight trend towards decreased confidence in the professionalism of nursing staff. This somewhat contradicts the findings that family members value nurses’ friendliness and ability to meet patients’ basic care and pain management needs. However, this may be partly explained by the prevalence, during the period of the study, of substitute nurses who had less knowledge about caring for older cancer patients than permanent staff might have had [24, 38].

### Factors explaining satisfaction with care

The results show that family members evaluated patient care more negatively than patients did and that overall satisfaction with participation in care was low among both patients themselves and their family members. Family members of older cancer patients have described having the feeling that only the patient gets noticed [12, 40]. The involvement of family members is crucial to enabling patients to cope during their treatment and to clarifying the information that is received [11, 12]. However, both patients and family members reported a lack of involvement in decision making and that they did not receive enough information, to the extent of feeling uncertain about the treatment being given [41, 42]. For the latter, it is essential to use everyday language rather than medical language [7, 11, 12].

### Limitations

The results of this study should be interpreted with caution. Firstly, data were collected from a single cancer hospital and cannot be generalized. Secondly, the cross-sectional design of the study means that the findings indicate the situation at a specific moment in time. Thirdly, the data were collected through a self-reported questionnaire, and it is possible that questions may have been misunderstood. It is also worth noting the relatively high level of education among respondents, which may have had an influence on the results.

## Conclusions

In this study, we demonstrated that patients and family members were satisfied with their care. The care was described as friendly, respectful, and based on patients’ needs. Nevertheless, patients and family members perceived that the goal of care was not fully clarified, and opportunities to participate in care were limited. Family members perceived that the atmosphere in which care was given was busy; on the ward, there were not enough nursing professionals to listen to patients’ worries, feelings about their health, or fears.

It is important that older cancer patients and their family members receive friendly, respectful, individual care based on their needs and hopes and that they can rely on professionals. Further studies are needed to identify the necessary competencies for the holistic care of older cancer patients and their family members. Management needs to acknowledge that providing quality care requires adequate resources: additional education is needed, and hospitals should give nurses opportunities to use their clinical expertise in the complex care of older cancer patients and their family members.

## Data Availability

The datasets generated and/or analysed during the current study are not publicly available due to the sensitive nature of the research but are available from the corresponding author on reasonable request.

## Abbreviations

RHCS: Revised humane caring scale
ANCOVA: Analysis of covariance
WHO: World health organisation
IOM: Institute of medicine
